# Correction

**DOI:** 10.1111/cas.15016

**Published:** 2021-07-02

In an article^1^ titled “Upregulation of glucocorticoid receptor‐mediated glucose transporter 4 in enzalutamide‐resistant prostate cancer” by Seiji Hoshi, Satoru Meguro, Hitomi Imai, Yuta Matsuoka, Yuki Yoshida, Akifumi Onagi, Ryo Tanji, Ruriko Honda‐Takinami, Kanako Matsuoka, Tomoyuki Koguchi, Junya Hata, Yuichi Sato, Hidenori Akaihata, Masao Kataoka, Soichiro Ogawa, and Yoshiyuki Kojima, the following errors were published:

- The first name of the sixth author was misspelled. It should be “Akifumi” instead of “Akihumi,” so it reads “Akifumi Onagi.”
- The graph was missing in Figure 6C. The correct figure is presented below.

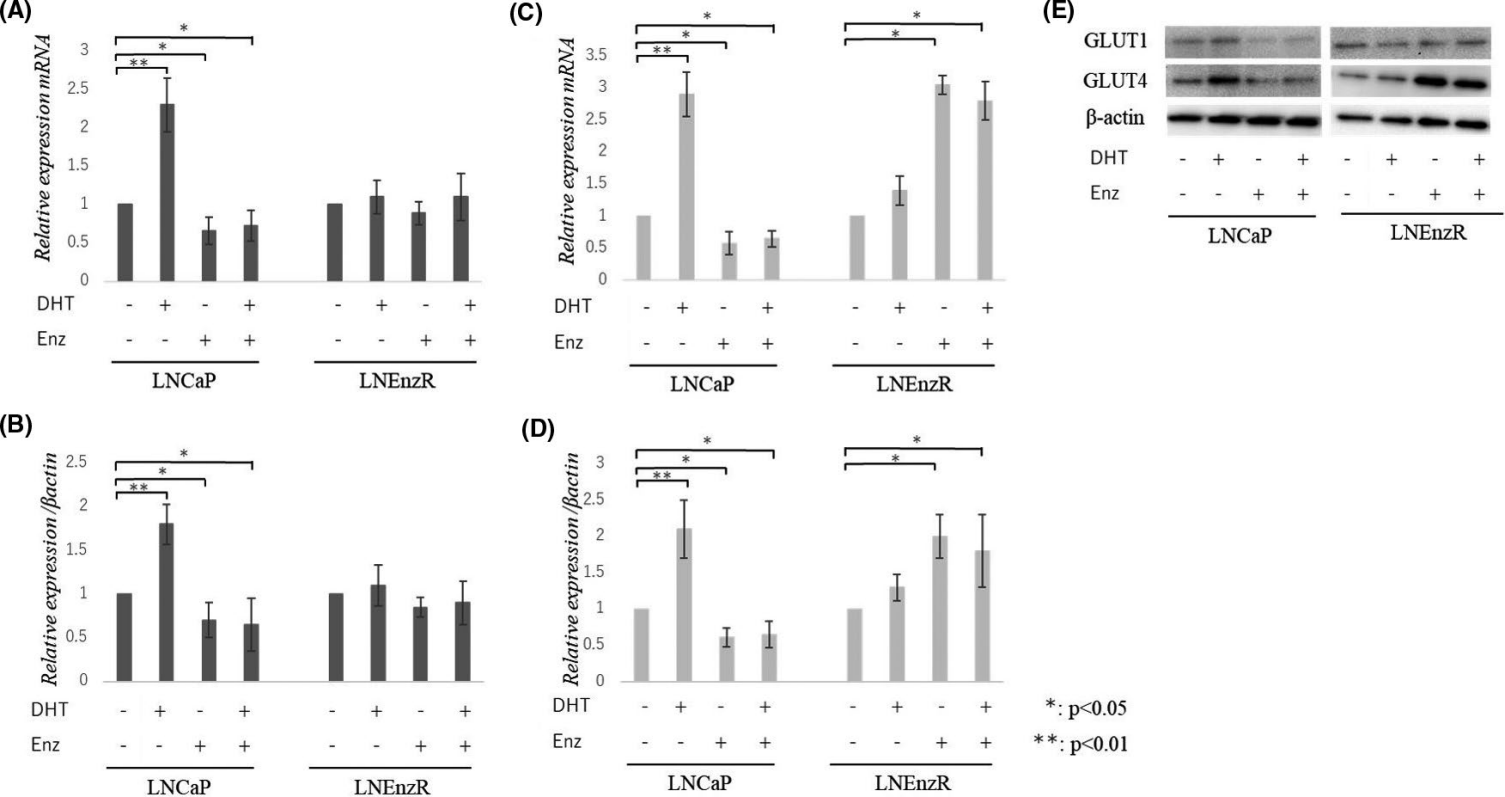

The authors apologize for the errors.
